# Women’s relative status and childbearing intentions: Empirical evidence from Iran

**DOI:** 10.1371/journal.pone.0195428

**Published:** 2018-04-12

**Authors:** Maryam Moeeni, Arash Rashidian, Akbar Aghajanian

**Affiliations:** 1 Social Determinants of Health Research Center, Isfahan University of Medical Sciences, Isfahan, Iran; 2 Health Management and Economics Research Center, Isfahan University of Medical Sciences, Isfahan, Iran; 3 Department of Health Management and Economics, School of Public Health, Tehran University of Medical Sciences, Tehran, Iran; 4 Department of Sociology, FSU-UNC, Fayetteville, North Carolina, United States of America; Karolinska Institutet, SWEDEN

## Abstract

Childbearing intentions are primary predictor of childbearing behaviors, particularly in low fertility societies. This study examined the role of relative status of women in childbearing intentions in Iran where fertility has been declining since 1986 and it has been around the replacement level during the last two decades. Data from the 2010 Iran’s Multiple Indicator Demographic and Health Survey (IrMIDHS) were used to estimate the effect of relative status of women on intention to have more children among women with one child and those with two children. The results showed modest effect of relative status of women on future childbearing intentions at both parity one and two controlling for socioeconomic and demographic factors. One implication from this finding is that within low fertility regimes where fertility level is around or below replacement level, the relative status of women is no more as important determinant of childbearing intention as in situations of high fertility regimes. This interpretation is consistent with the fact that most of the studies showing strong effect from relative status of women on childbearing are based on data from the situations where fertility level has been at the pre-transitional level.

## Introduction

Patterns and trends in fertility changed in Iran from 1970 to 2012. Despite the modest decline during the 1970s, the total fertility rate (TFR) rose rapidly in the first half of the 1980s. However, starting in 1986, Iran experienced the most significant decline ever recorded worldwide. Total fertility rate (TFR) dropped from 6.5 in mid-1980s to approximately replacement level fertility in 2000. During the decade of 2000–2010, the TFR has been below the replacement level on the average with some variation across provinces [1]. Based the most recent data and estimates currently Iran’s TFR ranks low among the countries of the Middle East. Public concerns and official debates have been raised about continued decline in fertility leading to an old age structure and its social and medical issues [1–8].

Within the context of this political and scientific debate and availability of national fertility data from surveys and censuses, an extensive literature has developed on the fertility transition and path to fertility decline [1–6]. While a number of these studies refer to the status of women as an important factor in transition to low fertility, studies focusing on this topic and particularly the role of relative status of women in this low-level fertility regime are very limited [4, 5]. The availability of the 2010 Iran’s Multiple Indicator Demographic and Health Survey (IrMIDHS), provides a great opportunity for analyzing the impact of relative status of women as a determinant of childbearing intention in the context of the low fertility environment. In this paper we analyze childbearing intention of currently married women within a sequential framework and examine the impact of relative status of women.

## Analytical framework

Empirical research supports the association between intended and actual childbearing in low fertility countries [7–13]. The analytical framework in this paper draws on the association of childbearing intention and actual child bearing in the context of preference theory [14, 15]. Specifically, this assumption suggests that there is an association between reproductive preferences and observed fertility [16]. Given this strong association, couples’ fertility preferences are reflected in their current expressed childbearing intention [14, 15, 17]. Hence our analysis in this paper is centered on childbearing intentions of married women.

The theoretical and empirical literature on childbearing intentions has documented the role of sequential decision making and the existing level of fertility in relation to fertility behavior. Specifically, couples make decision about having more children based on the current number of children and their prospective life expectancy [13, 18–25]. Accordingly, our analysis will utilize a sequential approach which includes not only the current number of children but their sex composition. The latter factor is very important in the context of the Iranian culture where son preferences has a long tradition and having at least a son, has been an important factor in higher fertility.

A longstanding argument exists in regard to the role of status of women and their childbearing decision making. At the individual level, variables such as education or working status of women have been considered as measures of women’s status. However, the effect of these variables would disappear once the economic variables are included in the analysis. This suggests that, the economic characteristics of women per se are not reflection of their status. Hence, researchers have focused on measuring the status of women as a relative concept [26, 27]. Conceptually women’s relative status draws from the bargaining power she has in joint decision-making and in relation to her husband. This relative bargaining power is different from macro-level concepts such as women’s empowerment, women’s autonomy and women’s agency measured at societal and community level [26–30]. The women’s relative status is associated with their ability to engage in alternative roles to childbearing and childrearing [34]. Previous research has shown that relative status differentials of women in the household, draws from age and educational differences between women and men [26–29]. Accordingly, we hypothesize that in the context of the Iranian society; women younger than their husbands and those with less education than their husbands, have less engagement in alternative roles to childbearing and hence have higher fertility intentions and preferences.

Economic theories of childbearing and the extensive empirical research based on these theories have documented the role of couples’ economic standing in childbearing decision and intentions. Starting with Becker’s seminal work, known as the ‘unitary model’, economic researchers have documented the impact of economic status on fertility [31, 32]. The economic linkage between fertility and economic status is through children’s cost consideration, including opportunity cost of mother, and cost of high quality of children and allocation of household resources [31–33]. In this paper we use the availabe measures of economic status of couples in analyzing women’s childbearing intentions to control for the effect of economic status of the couples.

A key feature of preference theory is that the variables determining childbearing preference may have different impact based on the social context in which they occur [15]. This observation on contextual effect of the community or the social milieu in which the individual lives, has been viewed as a socio-psychological as well as social ecological phenomenon which does not only affect all behaviors and attitudes including childbearing behavior and preferences [34–36]. The concept of reference group can be used appropriately to explain the mechanism through which community characteristics affect the individual childbearing [34, 35]. Broadly, this means that individual self-perception and behavior conforms partly to the standard observed in the community and this standard is determined by the community characteristics such as the fertility patterns and level. Hence our analytical model includes a measure of provincial level of fertility expected to affect the women preferences, beyond and above the individual determinants of childbearing intentions.

## Materials and methods

### Data and study population

The data for this research is drawn from the 2010 Iran’s Multiple Indicator Demographic and Health Survey (IrMIDHS). This is a large survey based on a stratified random sampling design conducted by Ministry of Health and Medical Education in consultation with the Statistical Center of Iran (for details see Ministry of Health and Medical Education, Year 2010). Primary sampling units were selected randomly in the rural and urban areas of each of 30 provinces. Since 2010, Iran has 31 provinces. In that year, the Alborz province was divided from the Tehran province to form the 31^st^ province of Iran. However, 2010 IrMIDHS surveyed the Tehran and Alborz as one province and did not collect separate data for Tehran and Alborz. The sampling frame was based on the 2005 census. As a result, a total of 31350 households were contacted of which 30960 participated in the survey. In the total of 30960 households, 22526 ever-married women aged 15–49 years were interviewed among them 19099 were married at the time of interview and had reproductive potential. After excluding households reporting to have step children or adopted children, extended and polygamous households, we reached a sample of 17620 currently married women of which 12530 had answered the question about their childbearing intentions and the rest had missed the question. Among the women who had responded to the question about childbearing intention, 8045 had one child or two children and were able to have more children at the time of survey. The data from these women were utilized in this study. All respondents of IrMIDHS had provided verbal informed consent before interview. The ethics board of Isfahan University of Medical Sciences approved our study procedure. The ethical code is IR.MUI.REC.1396.2.101.

### Measures

#### Dependent variables

A woman’s childbearing intention is coded based on the question ‘Do you intend to have any other children?’. A dichotomous variable was constructed based on the three possible responses. We wanted to focus on certainty of having another child. Hence, for women who responded “yes” the question was coded “1”. Those responding “no” or “I cannot say/I am not sure” were coded as “0”.

#### Independent variables

Indicators of women’s relative status were constructed based on husband-wife differences in age and education. Two variables were constructed based on age and level of education of wife and husband: (1) age differential and (2) education differential, in favor of husband. Our expectation is that both of these variables reflect the authority and relative status of women in making decision about their roles. Specifically, the lower these two variables, we assume the higher is the authority and relative power of wife in decision making in the context of the household behavior and roles outside of the household.

There is a long tradition of son preferences and desire for at least one son among parents in Iran [37]. It is possible that even within the current low fertility regime, this variable affects the decision of couples regarding intention to have more children. Hence, data on the sex composition of current children was used to construct the children-sex composition variable. The categories for sex composition of children are: only son, both son and daughter, only daughter. Also, age of the youngest child of the women was used as a determinant constraint on intending another child.

Collection of reliable income data has been very difficult in the context of the Iranian society due to strong underreporting. Hence, for the purpose of measuring economic status as determinant of childbearing intentions we used husband’s education, home ownership of the couple and age of the husband. We assume that men with more education, particularly college level education, have a higher income as they may have professional jobs and are able to utilize the opportunities in the formal economy. On the other hand, men with no education or low education are more probable to work in manual labor, low wage service sector, and as agricultural laborers. This variable has three categories: less than high school diploma, high school diploma, and some college.

Homeownership particularly in urban areas is an important measure of better economic standing for couples in Iran particularly for those living in highly populated cities. Migration and urban-based economic development has created a continuous pressure on housing and housing prices in recent decades [38]. Hence, renter couples are in much lower economic status than home owners. We use renting status as compared to ownership status as an additional measure of economic standing of couples. We also control for the age of husband as a measure of experience and time in the labor market.

We measure the current fertility norm of the community by total fertility as of 2010. As discussed in the previous section, we hypothesize that the fertility norm in the community has its own independent effect on childbearing intentions and preferences of the individual. Ideally, availability of contextual data at small community level such as villages or townships, would best fit the purpose of this hypothesis. The data for this variable was obtained from the Statistical Center of Iran. However, the data had been released at provincial level. In most cases, Iranian provinces are homogeneous culturally, geographically and economically.

### Data analysis

We used logistic regression analysis to determine the effect of independent variables on probability of decision to have more children. The statistical analysis was conducted for women with one child and women with two children separately. We ran one model with individual level variables and a second model including the provincial level of fertility for each group. The statistical analysis was conducted with Stata version 11 and the results were shown in Tables one through three.

## Results

### Descriptive characteristics

Table 1 shows the distribution of the dependent and independent variables for the 8045 currently married women with one or two children. About 38% of the women want more children. The mean for educational difference between husband and wife seems to be small, but there is a large variation in this variable. The mean age differential is about 4.7 and the variable has a relatively large variance. About 33% of women have only daughters and this group is expected to have a much higher probability to express interest in having more children in future. In contrast to these women, 48% of women living in rental housing are expected to express less intention for further childbearing.

**Table 1:** Summary statistics for the variables.

% women wanting more children	37.5
Mean Husband-Wife Age differential	4.7 (4.5)
Mean Husband-Wife Education differential	0.2 (3.7)
Mean Age of the youngest child	4.8 (5.2)
Sex-composition of current children:	
% son only	40.1
% Daughter only	32.8
% A son and a daughter	27.1
Mean Husband Age	35.1 (7.4)
Husband Education:	
% less than High School	53.6
% High School	28.4
% Some College	18.0
Homeownership:	
% Owner	51.6
% Renter	48.4
Provincial Total Fertility	1.8 (0.4)

Standard deviation in prentices

To follow our sequential decision-making framework, we analyzed the data first for women who had one child at the time of the survey. The logistic regression results are reported in Table 2. Among these women 60% reported “yes” they intend to have a second child. Both age and education differentials in favor of husband, have significant but modest effect on intention for further childbearing among women with one child. While our main hypothesis from these results is supported, we also note some interesting findings from the control variables. For example, we observed that sex of the previous child is more important than relative status variables. The women who have one daughter, had much more probability to report intention for further childbearing in future, in contrast to women with one son, controlling for the age of the current child. This finding points to the importance of son preferences even in the contemporary low fertility normative order.

**Table 2:** Logistic regression results for desire to have more children among women with one living child.

Variables	1	2
Husband-Wife Age differential	0.037**	0.034**
Husband-Wife Education differential	0.059**	0.057**
Age of the youngest child	-0.051	-0.041***
Sex-composition of current children:		
Son	Reference	Reference
Daughter	0.191**	0.178*
Husband Age	-0.077***	-.071***
Husband Education:		
less than High School	Reference	Reference
High School	-0.422***	-0.383***
Some College	-0.591***	-0.570***
Homeownership:		
Owner	Reference	Reference
Renter	-.378***	-0.369***
Provincial Total Fertility		1.025***
Goodness of fit tests:		
Hosmer-Lemeshow chi 2	23.11**	14.57
Pearson chi2	3793.73	3905.18
Sample size	3852	3852

***: P-value<0.001,

**: P-value<0.01,

*: P-value<0.05

Regarding the economic variables, first controlling for age of husband, the higher the education of husband, the less intention for a second child. In a similar manner, women who live in rental housing, show much less probability to intend to have a second child. The second model in Table 2 adds total fertility of the province as a measure of fertility norm to the individual-level variables. The results are consistent with our hypothesis drawn from Hakim theory that the norm of the community has its own independent effect on childbearing intention. Women with one child, who live in higher fertility provinces, have higher probability to intend for a second child controlling for all the individual determinants of fertility intention.

Table 3 reports logistic regression results for childbearing intention of women with two children. Among these women, 16% intend to have a third child. However, this measure varies by individual and provincial characteristics. Like women with one child, the relative status variables have significant but modest effect on intention for further childbearing. These findings are comparable across the two group of women at parity one and parity two. Hence, regardless of parity, women who are married to older and more educated men, seem to be more inclined to have a third child in future. We also find that women with two daughters have a much higher probability to intend more children in future, as compared to women with two sons or a son and a daughter. Again, we continue to see the role of son preferences in relation to childbearing intentions.

**Table 3:** Logistic regression results for desire to have more children among women with two living children.

Variables	1	2
Husband-Wife Age differential	0.055*	0.048**
Husband-Wife Education differential	0.060**	0.060**
Age of the youngest child	-0.51***	-0.025
Sex-composition of current children:		
son	Reference	Reference
Daughter	0.478***	0.523***
One daughter one Son	-0.341**	-0.365***
Husband Age	-0.111***	-0.088**
Husband Education:		
less than High School	Reference	Reference
High School	-0.351**	-0.367**
Some College	-0.435**	-0.543***
Homeownership:		
Owner	Reference	Reference
Renter	-0.261**	-0.203*
Provincial Total Fertility		1.191***
Goodness of fit tests:		
Hosmer-Lemeshow chi 2	17.64*	6.43
Pearson chi2	3976.12	3957.80
Sample size	4193	4193

***: P-value<0.001,

**: P-value<0.01,

*: P-value<0.05

The effect of economic variables, husband education and renter status, are consistent with our expectation. The higher the educations of husbands, the lower the probability of intention for a third child and probably more focus on the quality of the existing ones. The renter women show much lower preference for further childbearing as couple’s income and housing status are strong constraints on quantity and quality of children.

When we include the total fertility of the province as a contextual variable in the model predicting the childbearing intention of women with two children, we find that the coefficients for individual variables change very little and fertility level at province has the strong effect on women’s childbearing intentions. The women living in provinces with higher fertility level, have more probability of expressing desire for additional children. Again, this suggests the strong role of community fertility norm on individual intention independent of the individual characteristics.

## Discussion

This study was focused on investigating the role of relative status of women on their childbearing preferences measured by intention for further childbearing in future. Contrary to our expectation, the relative status of women did not show strong effect on childbearing intention neither for women at parity one nor for women at parity two. One explanation for this modest impact of relative status of women maybe that this variable is not as determinantal in such a low fertility regime—below replacement level in many provinces—as compared to when fertility was at pre-transitional level. This explanation is supported by the fact that most of the studies showing strong effect for relative status of women are based on data from the time and the countries at pre-transitional fertility level [26, 28, 30, 39, 40].

Our multivariate analysis of data showed that despite significant changes in the attitude of young cohorts of women about children and family formation in Iran, the tradition of son preference continues to rule in childbearing decision making. This persistence of son preference tradition is strong despite the constraints on childbearing intention from expensive housing. Similar to the persistence of son preferences, the high fertility norms of the community, continues to pressure women for further childbearing, independent of their individual characteristics.

This study has several limitations. The data lacks information on men’s childbearing intentions because only women’s intentions were asked in IrMIDHS. Moreover, it was difficult to gain access to precise data on women or family economic status. Thus, economic measures are proxies and do not fully capture the economic status of women. Therefore, our findings offer only tentative conclusions regarding the importance of couple’s economic status on women’s childbearing intentions. The other limitation of the study was considerable missing data related to women’s childbearing question.

Despite these limitations, the study results provided significant insights to the analysis of fertility intentions of women in a low fertility regime country in the Middle East, where women have been significantly increased their education and over 70% of the population is living in urban areas.
